# Role of Mitanin community health workers in improving complementary feeding practices under scaled-up home-based care of young children in a rural region of India

**DOI:** 10.1186/s12887-023-03993-4

**Published:** 2023-04-13

**Authors:** Samir Garg, Mukesh Dewangan, Kavita Patel, C. Krishnendhu, Prabodh Nanda

**Affiliations:** State Health Resource Centre, Chhattisgarh, Raipur India

**Keywords:** Young child care, Home based child care, Complementary feeding, Community health workers, Infant and young child feeding, Integrated management of child illness, Mitanin, ASHA, India

## Abstract

**Background:**

A large proportion of young children in developing countries receive inadequate feeding and face frequent infections. Global research has established the need for improving feeding practices and management of child illnesses. Interventions involving home visits by community health workers (CHWs) for caregiver education have been attempted in many countries. Indian government rolled out an intervention called home-based care of young children (HBYC) in 2018 but no studies exist of its scaled-up implementation. The current study was aimed at assessing the coverage of HBYC in Chhattisgarh state where it has been implemented through 67,000 rural CHWs known as Mitanins.

**Methods:**

This cross-sectional study was based on a primary household survey. Households with children in 7–36 months age were eligible for survey. A multi-stage sample of 2646 households was covered. Descriptive analyses were performed and key indicators were reported with 95% confidence intervals. To find out the association between caregiver practices and receiving advice from the CHWs, multivariate regression models were applied.

**Results:**

Overall, 85.1% children in 7–36 months age received at least one home visit from a CHW within the preceding three months. Complementary feeding had been initiated for 67% of children at six months age and the rate was 87% at eight months age. Around one-third of the children were fed less than three times a day. Around 41% households added oil in child’s food the preceding day. CHWs were contacted in 73%, 69% and 61% cases of diarrhea, fever and respiratory infections respectively in children. Among those contacting a CHW for diarrhea, 88% received oral rehydration.

The adjusted models showed that receiving advice from CHWs was significantly associated with timely initiation of complementary feeding, increasing the frequency of feeding, increasing diet diversity, addition of oil, weighing and consumption of food received from government’s supplementary nutrition programme.

**Conclusions:**

Along with improving food security of households, covering a large share of young children population with quality home visits under scaled-up CHW programmes can be the key to achieving improvements in complementary feeding and child care practices in developing countries.

**Supplementary Information:**

The online version contains supplementary material available at 10.1186/s12887-023-03993-4.

## Background

Appropriate feeding practices are essential for the survival, growth and development of infants and young children. These feeding practices are known collectively as infant and young child feeding (IYCF) [1, 2]. The recommended practices include timely initiation of breastfeeding, exclusive breastfeeding till six months age, continuation of breastfeeding till two years age, timely introduction of complementary foods, sufficient meal frequency and portion size, diversity in diet, hygiene and responsiveness to feeding cues [1–3]. Optimal IYCF can prevent a significant share of deaths in children under five years of age [4, 5]. The period from birth till 2 years of age is a critical window period to promote health and development and prevent stunting [1–5].

Global research shows that IYCF indicators and caregiver practices are at suboptimal level [3, 6–8]. Globally, around 44% infants 0–6 months old are exclusively breastfed [3]. In many low- and middle-income countries (LMICs), less than a fourth of infants in 6–23 months age met the criteria of dietary diversity and feeding frequency that are appropriate for their age [7]. The delay in timely introduction of complementary feeding is a problem across most regions of the globe. About one third of infants globally in 6–8 months age did not receive solid, semisolid, or soft foods [7]. The gap in IYCF practices is wider in LMICs including India. Malnutrition is a major contributor to disease burden and more than half of the global deaths in children younger than 5 years of age are attributable to under-nutrition [9]. A big share of such burden is concentrated in LMICs including India [9]. In 2015–16, 38% children under age of five in India were reported as stunted, 45% were not exclusively breastfed for six months as recommended and 54% of children aged 6–8 months did not receive any solid or semi-solid foods [10].

Apart from the gaps in feeding; malaria, diarrhea and respiratory infections have a huge bearing on survival and growth of infants and young children [11]. Among children 1–59 months of age in India, pneumonia and diarrhea respectively contributed to 30.6% and 21.3% of child deaths in 2015 [12]. The incidence of diarrhea among children over the preceding two weeks of in an Indian national survey was 9.2%, while 73.2% children had an episode of fever or respiratory infection [10]. Undernourished children are at significantly higher risk of frequent infections, severe disease and mortality [13]. A vast number of children in India and other LMICs are trapped in a vicious cycle of under-nutrition and infections [13]. It is therefore important to simultaneously address the gaps in feeding along with preventing, detecting and treating the infections among young children.

In terms of solutions, research has established the importance of nutrition education to improve knowledge of caregivers about IYCF practices [14]. Community health workers (CHWs) have been found to be effective for improving IYCF practices in communities [15]. Many Countries have also implemented integrated management of child illnesses through CHWs [16, 17].

India has now a workforce of around a million CHWs known as the Accredited Social Health Activists (ASHA). In 2011, an initiative on home based newborn care (HBNC) was rolled nationally through ASHA CHWs. HBNC was based on CHWs making a series of structured home visits to counsel caregivers on newborn care and identify sickness in newborns. Subsequently, there was a realization that young children beyond the newborn age also need to be covered with structured home visits and ASHA CHWs should be the vehicle to do so [18]. A pilot intervention known as HBNC-plus was launched by the governments in multiple states in 2013. An evaluation of this intervention showed that although the coverage of young children was greater in the intervention arm, it did not result in improved IYCF outcomes [18]. Another study of the same intervention showed the intervention areas achieved greater coverage of iron folic acid (IFA) supplementation and growth monitoring but not much difference was found in breastfeeding and complementary feeding [19]. Those studies did not shed much light on the identification and management of illnesses in children.

Most of the studies on IYCF in India have been on small scale interventions and largely focused on the newborn age group. There is a need to understand the changes achieved through scaled up home visits by CHWs for the children above 6 months age. Making home visits for infants and young children is now an integral part of the nationwide ASHA CHW programme in India. It received a major boost in 2018 when the central ministry of health launched a nationwide programme known as the Home-Based Care of Young Children (HBYC). HBYC integrates essential components of young children’s care – feeding, prevention and management of child illnesses and early childhood development. HBYC is expected to be delivered by ASHA CHWs through quarterly home visits.

In India, Chhattisgarh state was a pioneer in developing a scaled-up CHW programme [20]. The programme was started in 2002 [20]. The state had a population of 29 million in 2020 and around three-fourth of that lived in rural areas. There were 67,000 CHWs known as Mitanins who covered the rural population. The average population covered by a Mitanin was around 330. In rest of India, the average population per ASHA was around 1000. Each Mitanin belonged to the habitation she worked for [21]. Mitanins were selected by the communities they serve through a process of community meetings facilitated by civil-society actors working in partnership with the government. The programme had a dedicated support structure for Mitanins. Each cluster of 20–25 Mitanins was supported and supervised by a Mitanin-Trainer who belonged to the local area. Most of the Mitanin-Trainers had been selected from amongst the Mitanins of the concerned clusters. Mitanins had received around 100 days of residential training divided across around twenty modules. In addition, each Mitanin received two days of field-based training each month. Making home visits was included as a key task in work of Mitanins. Mitanins started as honorary workers but as their workload grew, task-linked payments were introduced including for home visits focused on child care [21].

Mitanins are generalist or multipurpose CHWs and they play multiple and wide-ranging roles [21]. Their role is not restricted to any particular disease or target-group (e.g., children). They are expected to work on a broad set of issues under preventive and promotive health in addition to detection and treatment of illnesses and making appropriate referrals [21]. Mitanins work on many primary care issues including malaria, tuberculosis, maternal and newborn care [22–25]. They also engage in inter-sector action for social determinants of health (SDOH) including malnutrition and gender-based violence [26–30]. Mitanins have been recognised as a leading example of CHWs acting as socio-political actors demanding accountability from government and acting as agents of social change [26–30].

Chhattisgarh was also the first state to integrate IYCF and management of child illnesses in the curriculum and work of CHWs on a statewide scale. Mitanins were trained on essential messages on infant and child feeding in 2002 and they received a full-fledged training on IYCF in 2009. Mitanin CHWs were trained to provide advice for cold and cough in children in 2003 and to treat diarrhea using oral rehydration solution (ORS) in 2005. In 2010, an independent evaluation showed that Mitanin programme was effective in achieving significant improvements in breastfeeding and reducing childhood stunting [31]. The current study was aimed at assessing the coverage of HBYC through Mitanins in rural Chhattisgarh in 2020. The main aspects of HBYC covered under this study included complementary feeding and child illnesses. The specific objectives of the study were:

- To assess the coverage of home visits and advice received from CHWs under HBYC for the age groups 7–12 months, 13–24 months and 25–36 months
- To assess the complementary feeding practices in families and their association with advice received from CHWs

## Methods and materials

This is a cross-sectional quantitative study based on a primary household survey. The eligible households were those with children in age of 7 to 36 months. The households were further sub-divided in three age-groups—those with a child 7 to 12 months, 13 to 24 months and 25 to 36 months age.

Sampling: For a confidence level of 95% and a desired precision of 5%, the sample size required was calculated as 385 eligible households. In order to account for a multi-stage sampling design, the sample requirement was increased by 50% to 578 households. The objective was to find out the key indicators for each of the three age-groups. The total number of households to be interviewed was decided so that at least 578 children in the age group of 7–12 months get covered. This ensured that there were enough children of the other two bigger age-groups.

The study selected a sample representative of eligible rural households in the state. The state has a total of 146 rural administrative units, known as blocks. Each block has around 350 to 500 habitations. Two habitations were selected from each rural block using systematic random sampling. Thus, 292 rural habitations got selected. The size of a habitation ranged from 30 to 80 households. In each selected habitation, all eligible households were to be surveyed. The survey was able to cover a total of 2646 households. It included 703, 1136 and 807 households with a child in the 7–12 months, 13–24 months and 25–36 months age-groups respectively.

Data collection: The survey was conducted in the month of November 2020. A semi-structured pre-tested questionnaire was used for interviewing the caregivers. Data was collected on the socio-demographic characteristics, complementary feeding practices by the family, supplementary nutrition received for children under the Integrated Child Development Services (ICDS) programme of government, weighing, home visits by Mitanin CHWs for HBYC and kinds of advice provided to caregivers, occurrence of illnesses in children and the role played by Mitanin CHWs in addressing the illnesses.

For assessing the HBYC home visits, the period was of three months preceding the survey. The questions on food consumed by each child were for the day preceding the survey. The types of foods covered in the survey included six types of foods – cereals, pulses, eggs/ meat/fish, vegetables and fruits. To reduce the possibility of recall bias, the question on age of initiating complementary feeding was limited to children in 7–12 months age. For occurrence of illnesses in children, a recall period of 15 days was taken. The specific illnesses covered were diarrhea, cold and cough, fever and pneumonia.

The participants were informed about the purpose of the study and consent was obtained before data collection. Ethics approval was obtained from the Institutional Ethics Committee of the State Health Resource Centre.

The list of study variables is given in Table 1. Descriptive statistical analysis was performed using cross tabulations. Confidence intervals at 95% were computed for key indicators and reported in parentheses. The preliminary analyses involved comparing the practices of households who received the relevant advice from CHWs and those who did not. The significance in the above comparisons was found out using the chi-square for proportions and the t-test for the means. For finding out the association between key complementary feeding (CF) practices and CHWs' advice while controlling for the likely confounders, multivariate analyses were performed by applying linear and logistic regression models.

**Table 1:** List of variables in the study

Variable Name	Variable Description	Category
Age of child	Age of child in months	Continuous variable
Sex	Sex of child	Male
		Female
Social Group	Social group (caste) of mother	Scheduled Tribes
		Scheduled Castes
		Other Backward Classes
		Others
Mother's Education	Education category of mother	8^th^ standard and above
		5-7^th^ standard
		1-4^th^ standard
		No education
Mother's age	Mother's age in years	Continuous variable
Family size	No. of members in the family	Continuous variable
Division	Name of the administrative/geographical division of the state	Raipur
		Durg
		Bilaspur
		Sarguja
		Bastar
Initiation of complementary feeding at 6 months	Whether complementary feeding was started at 6 months age (asked for children 7–11 months age)	Yes/No
Frequency of feeding	The number of times a child was given complementary feeding the previous day	Continuous variable
Advice received from Mitanin	Whether a particular advice was received by the respondent caregiver from Mitanin	Yes/No
Diversity of foods	No. of types of foods fed to child the previous day	Continuous variable
Addition of oil	Whether oil was added to child's food the previous day	Yes/No
Consumption of supplementary nutrition	Whether supplementary nutrition received from the government programme Integrated Child Development Services (ICDS) was consumed by child the previous day	Yes/No
Weighing	Whether the child's weight was measured at least once over the previous two months	Yes/No

The first adjusted model was for the dependent variable of initiation of CF at six months age. The independent variables included in the model were the socio-demographic characteristics (age of child, sex of child, age of mother, education attainment of mother, the household size, geography/region of residence) and whether advice was received on timely initiation of complementary feeding. The above variables were selected based on existing studies on determinants of CF in India [32, 33]. Similar models were applied one by one for the dependent variables of the other desired practices—frequency of feeding, diet diversity, addition of oil, consuming the supplementary food provided by government and weighing.

All the data analyses were conducted by using the STATA 15 software.

## Results

The socio-demographic profile of the households is given in Table 2.

**Table 2:** Socio-demographic profile of the sample and proportion of children who received a home visit from Mitanin in preceding 3 months

Demographic profile	Number	(%) (*N* = 2646)	Proportion of children who received home visit of Mitanin in previous 3 months (*N* = 2646) (%)
**Caste category (Social group)**			
Scheduled Tribes (ST)	901	34.1	86.5
Scheduled Caste (SC)	340	12.9	84.1
Other Backward Classes (OBC)	1,240	46.9	85.6
Others	165	6.2	76.4
**Family size**			
Less than 5 members	1,073	40.6	83.5
5 or more members	1,573	59.5	86.2
**Age of child**			
7 to 12 months	703	26.6	85.8
13 to 24 months	1,136	42.9	84.9
25 to 36 months	807	30.5	84.9
**Gender**			
Male	1,377	52.0	85.3
Female	1,269	48.0	84.9
**Mother’s education**			
8th standard or higher	1,844	69.7	85.2
5th to 7th standard	364	13.8	83.2
1st to 4th standard	103	3.9	87.4
No formal education	335	12.7	85.7
**Geographical Division**			
Raipur	522	19.7	84.7
Durg	539	20.4	86.6
Bilaspur	702	26.5	87.7
Surguja	459	17.4	82.6
Bastar	424	16.0	82.1
**All**	**2646**	100	**85.1**

### Coverage of home visits for HBYC

Overall, 85.1% (83.4%-86.8%) of the children in age group of 7–36 months had received a home-visit from a Mitanin in preceding three months (Table 2). The proportions of children receiving a home-visit from Mitanins were found to be similar across different categories (Table 2).

### Advice provided by CHWs under HBYC

The proportion of households receiving the various relevant messages from Mitanins during HBYC home visits is given in Table 3.

**Table 3:** Proportion of households receiving different HBYC messages from Mitanin CHWs

Advice/message	Proportion of households receiving HBYC messages from Mitanins—by age-group of children (% with 95% CI)			
	**7 to 12 months (*N* = 703)**	**13 to 24 months (*N* = 1136)**	**25 to 36 months (*N* = 807)**	**All (*N* = 2646)**
Starting complementary feeding at six months age	86.8 (84.3–89.3)	79.2 (76.9–81.6)	76.5 (73.5–79.4)	80.4 (78.9–81.9)
Continuing breastfeeding along with complementary feeding	88.9 (86.6–91.2)	85.3 (83.2–87.4)	79.4 (76.6–82.2)	84.5 (83.1–85.8)
Increasing frequency of feeding	89.5 (87.2–91.7)	89.5 (87.7–91.3)	89.5 (87.3–91.6)	89.5 (88.3–90.7)
Increasing diet diversity	76.5 (73.4–79.7)	76.6 (74.1–79.1)	75.6 (72.6–78.6)	76.3 (74.6–77.9)
Adding oil in complementary feeding	82.4 (79.5–85.2)	80.8 (78.5–83.1)	82.4 (79.8–85.0)	81.7 (80.2–83.2)
Feeding supplementary food received from ICDS	88.8 (86.4–91.1)	86.7 (84.7–88.7)	87.5 (85.2–89.8)	87.5 (86.2–88.8)
Continuation of feeding during illness	80.9 (78.0–83.8)	80.1 (77.8–82.4)	79.6 (76.8–82.3)	80.2 (78.6–81.7)
Giving ORS for diarrhea	81.8 (78.9–84.7)	80.0 (77.7–82.3)	83.0 (80.4–85.6)	81.4 (79.9–82.9)
Giving IFA supplementation	66.6 (63.1–70.1)	70.5 (67.9–73.2)	72.4 (69.3–75.5)	70.0 (68.3–71.8)
Regular weighing	83.1 (80.3–85.9)	80.0 (77.7–82.3)	80.8 (78.1–83.5)	81.1 (79.6–82.6)
Hand-washing for feeding	90.2 (88.0–92.4)	87.5 (85.6–89.4)	85.5 (83.1–87.9)	87.6 (86.3–88.9)
Psycho-social stimulation	77.5 (74.4–80.6)	73.4 (70.8–76.0)	74.7 (71.7–77.7)	74.9 (73.3–76.6)
Immunisation	90.6 (88.5–92.8)	89.5 (87.7–91.3)	88.0 (85.7–90.2)	89.3 (88.2–90.5)

During HBYC home visits, Mitanins assessed 67.6% (65.8–69.4) of the children for feeding provided the previous day. They also assessed 70.8% (69.1–72.6) children for recent illnesses.

### Complementary feeding (CF) practices of households

#### Initiation of Complementary Feeding

The distribution of infants according to the age at which CF was started is given in Table 4.

**Table 4:** Age at which complementary feeding was initiated

Age of starting CF	Proportion of children (7–12 months) (N = 703) (% with 95% CI)
At 6 months	67.3 (63.5–71.0)
At 7 months	19.7 (16.9–22.5)
At 8 months	3.3 (2.3–4.6)
At 9 months	4.1 (2.9–5.4)
At 10 months	2.7 (1.8–4.5)
At 11 months	2.9 (1.9–4.2)

There were 33% children for whom the initiation of complementary feeding happened later than the age of six months.

### Frequency of complementary feeding

The frequency of CF was asked for the day preceding the survey. Overall, 37% children had received CF five or more number of times in a day, while those receiving CF 1–2 times a day were 36% (Table 5).

**Table 5:** Proportion of children according to frequency of complementary feeding received the previous day

Frequency of complementary feeding on preceding day	Proportion of children (% with 95% CI)			
	**7 to 12 months (*N* = 703)**	**13 to 24 months (*N* = 1136)**	**25 to 36 months (*N* = 807)**	**Total (*N* = 2646)**
1–2 times	43.7 (39.9–47.6)	33.3 (30.3–36.6)	32.1 (28.5–35.8)	36.1 (34.1–38.1)
3–4 times	30.6 (27.1–34.3)	25.7 (22.9–28.7)	25.0 (21.8–28.6)	27.0 (25.1–28.9)
5 or more times	25.7 (22.5–29.2)	41.0 (37.7–44.3)	42.9 (39.1–46.9)	37.0 (34.9–39.0)

### Food diversity in complementary feeding

The diversity of food consumed by children was asked for the day preceding the survey. The responses have been summarized in Additional File S1.

The proportion of children by number of six different types of foods (cereals, pulses, vegetables, fruits, eggs, meat/fish) is given in Table 6. It shows that 44% of children consumed 4 or more types of foods in a day. Around 15% children consumed 1–2 types of foods.

**Table 6:** Types of foods consumed by children the previous day

No. of different types of foods	7 to 12 months (*N* = 703) (%)	13 to 24 months (*N* = 1136) (%)	25 to 36 months (*N* = 807) (%)	Total (*N* = 2646) (%)
1	2.5 (1.6–4.1)	0.7 (0.4–1.5)	0 (0–0)	1.0 (0.7–1.5)
2	23.1 (20.0–26.4)	13.0 (11.1–15.1)	9.7 (7.8–12.0)	14.6 (13.3–16.1)
3	40.1 (36.5–43.9)	39.8 (37.0–42.8)	41.7 (38.2–45.2)	40.5 (38.6–42.4)
4	23.4 (20.3–26.7)	33.1 (30.4–36.0)	33.5 (30.3–36.9)	30.7 (28.9–32.5)
5	8.1 (6.2–10.4)	10.1 (8.5–12.1)	11.4 (9.3–13.8)	10.0 (8.9–11.2)
6	2.8 (1.8–4.4)	3.2 (2.3–4.5)	3.8 (2.6–5.4)	3.3 (2.7–4.1)

### Supplementary Nutrition under government programme

Under the Integrated Child Development Services (ICDS) programme of government, there exists a provision for supplementary nutrition for the age group covered in the study i.e., children between 6 months and 3 years of age. In Chhattisgarh, children of the above age group receive 750 g of ready-to-eat powder (consisting of wheat, sugar, gram, soya and oil) each week. Around 85.9% (84.3–87.5%) children had received the above supplementary nutrition in the week preceding the survey. Out of them, 61.6% (60.2%-63.1%) children had consumed the ready-to-eat powder on the day preceding the survey.

### Addition of oil in the food

It was found that 40.9% (39.1%-42.8%) families were adding oil in food during CF. This proportion was 44.5% (40.9%-48.2%) for the 7–12 months group, 40.0% (37.2%-42.9%) for 13–24 months age and 39.0% (35.7%-42.4%) for the 25–36 months age group.

### Weighing

According to the caregivers, 75.7% (72.4%-78.7%) of children had been weighed at least once during the previous two months. The proportion of children weighed was 79.0% (75.6%-82.4%) for the 7–12 months group, 74.2% (71.1%-77.3%) for 13–24 months age and 66.9% (64.0%-69.8%) for the 25–36 months age group.

### Illnesses in children and the role of CHWs in managing them

Around 29% of the children had at least one episode of illness over the 15 days preceding the survey. The most frequent illness reported was cold and cough (21.7%), followed by fever (7.6%) and diarrhea (6.2%).

Of the children who had an episode of diarrhea during preceding fifteen days, 73.4% (66.1%-80.5%) had contacted a Mitanin. Out of those who contacted Mitanins, 87.7% were given oral rehydration solution by the Mitanin and 12.5% were referred by Mitanins to higher facilities.

Of the children who had an episode of cold and cough during preceding fifteen days, 61.4% (57.8%-65.1%) contacted a Mitanin. Out of those who contacted Mitanins, 66.8% were advised to take home remedies.

Mitanins were contacted for 68.9% (61.8%-76.1%) of the fever episodes in children. Of those who contacted Mitanin for fever in the malaria-endemic areas, 79.2% (64.5%-93.9%) were tested for malaria by Mitanins (*n* = 57).

### Association between receiving CHW's advice and desired practices

Table 7 provides a comparison of the desired practices between households who received CHWs' advice and those who did not. It showed that the indicators of CF were significantly higher for those who received advice from Mitanins.

**Table 7:** Comparison of practices according to advice received from CHWs

Indicator	Received CHW's advice	Did not receive CHW's advice	Test	*p*-value
Complementary feeding started at six months age	59.2 (54.7–63.7)	42.1 (29.2–54.9)	Chi-square	0.014
Mean frequency of complementary feeding (on previous day)	3.1 (3.0–3.2)	2.6 (2.3–2.8)	t-test	< 0.001
Mean number of food types fed (on previous day)	3.4 (3.4–3.5)	2.8 (2.8–2.9)	t-test	< 0.001
Consumed supplementary nutrition received from ICDS programme	56.1 (54.0–58.0)	31.1 (26.1–36.1)	Chi-square	< 0.001
Added oil in CF	46.5 (44.4–48.6)	15.5 (12.3–18.7)	Chi-square	< 0.001
Weighing done (in last 2 months)	95.2 (94.3–96.2)	84.2 (80.2–88.2)	Chi-square	< 0.001

The results of applying the adjusted models for the determinants of the desired practices are given in Additional File S2. It showed that receiving the advice from CHWs was significantly associated with each of the six desired practices covered under the analyses.

## Discussion

This study presents the first evaluation of a statewide scaled-up programme of HBYC in India after the initiative's national roll-out in 2018. Except for a baseline study in Bihar state, no other studies are available on HBYC initiative after 2018 [34]. The proportion of relevant population that a programme covers is of crucial importance in achieving the desired outcomes at the population level. Our study in Chhattisgarh state found that 85% of rural children in 7 months to 3 years age group had received a HBYC visit from a Mitanin CHW in the preceding three months. The coverage rate was found to be similar for children in different ages within the above age-group. A study in multiple Indian states had reported a coverage rate of 39% by ASHA CHWs for children in age group of 3 to 15 months. A study in Odisha state found that while 90% of the households (with a child aged 6–24 months) had received a quarterly home visit from CHWs, only 32% had received any counseling on complementary feeding [35]. These studies attributed the poor coverage of nutrition counseling to over-burdened CHWs and absence of payment for this work [18, 35]. A study in Rajasthan had reported a coverage rate of 46% among the 3–15 months age children by the ASHA CHWs [19]. A study from Mali found that 54.6% households had received a visit from a CHW during the preceding 3 months [36]. The coverage rate of HBYC found in Chhattisgarh seems to be greater than the reported rate elsewhere. Around 70% of the households reported that Mitanin CHWs assessed the child's previous day's feeding and enquired about illnesses. The above feedback from the caregivers reflected a key dimension of quality of home visits by Mitanins.

This study found that complementary feeding had been initiated for 87% of children before completing 8 months of age. An earlier study on work of Mitanin CHWs in Chhattisgarh had shown a similar rate [31]. Two urban studies from India had reported that respectively 84% and 73% of children in 6–8 months age were receiving complementary feeding [33, 37]. The corresponding rate in a Ghana based study was 72·6% [38]. An intervention study in Palestine showed that the timely introduction of complementary feeding had improved from 71.5% to 87% through organized home visits [39]. In our study, 66% children had started complementary feeding at six months age. A study conducted in Karnataka state reported 40% of babies were started on complementary feeding at six months age [40]. A study conducted in Ethiopia showed 56.4 per cent of mothers introduced complementary feeding at six months [41]. Our study showed that receiving advice from Mitanin CHWs was associated with greater chances of timely initiation of complementary feeding.

Our study showed that receiving advice from Mitanin CHWs was associated with greater frequency of feeding. Around 51% children were fed five or more times in a day, while percentage of children receiving it three or more times was 78%. An earlier study of Mitanin programme in Chhattisgarh showed that 50% of children in 6–36 months were fed 3–4 meals per day [31]. This indicates that the frequency of feeding has improved in Chhattisgarh over the last decade. According to the available Indian and international studies, the minimum meal frequency was practiced for 49% to 86% children [33, 37, 39, 42].

Our study showed that receiving advice from Mitanins was associated with increasing diversity of foods in children's diet. Around 44% of children consumed four or more types of foods. Studies from other parts of India have reported minimum diet diversity (consuming four or more food groups) ranging from 33 to 67% [37, 42–44]. The proportion of children consuming eggs, fish/ meat and fruits was found to be poor in the current study. The poor consumption of poultry or meat has to be assessed in the context that around 82% of population in Chhattisgarh is non-vegetarian [45]. Chhattisgarh is one of the poorest states in India [46]. This underscores the need to strengthen the policies on food security to improve its impoverished population’s access to diverse foods.

This study also shed some light on consumption of supplementary nutrition of the Take Home Rations received for 7 month-3 years children from the government programme (ICDS). It showed that receiving advice from Mitanin CHWs was associated with increased chances of the above food being consumed by the children. While the ICDS programme had provided supplementary food for 86% of the children, the overall proportion of children actually consuming it the previous day was 51%. This indicates the need to explore other options of providing supplementary nutrition to this age group, including feeding hot cooked meals and providing crèches or daycare services. There may also be a need to strengthen and expand nutrition-supplementation and food security programmes like the ICDS.

Our study is one of the very few studies to collect information on addition of oil (or butter) to children’s food. The calorie density is an important aspect with respect to child feeding in this setting because widespread calorie and fat deficiency prevails in the diet of young children in India [47]. This aspect requires further attention in research on HBYC and complementary feeding in contexts such as India’s. In our study, 41% of households reported that they added oil in CF the preceding day. An earlier study in Chhattisgarh showed that one third of the families added oil to their child's food [31]. An intervention study in Pakistan showed that oil was added while feeding for 26% of children in the intervention group whereas it was 13% in the control group [48]. Although further improvements are needed in this practice, advice by Mitanin CHWs was found to be associated with greater chances of adding oil.

Regarding care seeking during an episode of illness, it was found that Mitanin CHWs were contacted in 73%, 69% and 61% of cases of diarrhea, fever and cold/cough respectively in children. Such coverage rates are significant considering the statewide scale of HBYC programme assessed in our study. Most of the existing Indian literature has examined the role of CHWs in managing child illnesses by interviewing the CHWs and is largely focused on assessing their knowledge [49–51]. Few existing studies in India have used responses of caregivers to examine the coverage of sick children by CHWs. A study in Karnataka had reported that 71.1% of diarrhea cases had received services through ASHA CHWs [40]. Another Indian study had reported that around one-third of respiratory-infection cases and one-fourth of diarrhea cases had received treatment from ASHAs [52]. Our study found that among those contacting Mitanin CHWs for diarrhea treatment, 88% received ORS. In an urban study in India, 32.5% of the diarrhea cases received ORS [53]. A study in Karnataka reported that 49.8% of diarrhea cases had received ORS [40]. Small intervention studies on CHWs in African countries have reported coverage rates of child illnesses ranging from 23 to 68% [54, 55].

Global literature cautions that merely deploying and training CHWs may not be sufficient to achieve the desired coverage and results [54, 55]. CHWs need to respected and supported by the larger health systems [55, 56]. CHW action is likely to be more effective when they are enabled to work on social determinants of health and nutrition and the necessary linkages are developed with other sectors and services [57]. Earlier Indian studies showed that ASHA based HBYC interventions were able to improve CHW competence but were not so effective in improving complementary feeding on the ground [18, 19]. The underlying reasons were the poorer payment and lower priority given for HBYC in comparison to other tasks of CHWs [18, 19]. Studies have found health systems factors such as poor support to CHWs, gaps in training and poor availability of medicines as the main factors impeding success [18, 54]. In this light, the positive findings of the current study hold significance for young child care in Indian and similar LMIC contexts. It shows that it is possible to achieve high coverage and improvement in practices through scaled up interventions through CHWs. The better density of CHWs in Chhattisgarh could have helped the high coverage under HBYC [21]. Another key factor could be related to the systems of training and supportive-supervision in the Mitanin programme. Mitanins received at least a week of residential training annually. It was supplemented each month by two days of practical training that focused on home visits. The supervisory cadre of Mitanin-Trainers played a key role in helping Mitanins in learning skills including by accompanying them during home visits. Further studies will be needed to find out if the ASHA CHWs in other states of India received similar kind of training and support. The inclusion of nutrition as a key health issue for CHW action and the programme’s emphasis on home visits in training and supportive supervision of Mitanins could have been facilitating factors [24, 27, 28, 31]. A recent study on workload of Mitanins has reported that they were working around 20 h per week and home-visits formed a fifth of it [21]. Mitanins enjoyed the trust of the families and communities they serve and that could have aided the uptake of their advice [27, 28]. The programme design allowed Mitanins to have a wide-ranging role. It enabled them to work substantially on the social determinants of health including the government programmes on food security [24, 27, 28].

Our study has several strengths. It was based on interviews of caregivers and not limited to responses from CHWs. It covered multiple dimensions of child feeding that often get ignored in CHW and child-health literature. It was able to examine the association between delivery of advice by CHWs and the change in child feeding practices. Further research is recommended to identify the factors that allow the Mitanin programme to achieve the desired coverage and effectiveness.

### Limitations of the study

The study is cross sectional. The data on practices of families presented here were as reported by the caregivers and not by actual observation. The existing studies have reported the household income as a relevant factor in complementary feeding but our study did not collect this data. The sample size was inadequate to find out the association of CHW intervention with appropriate care for child illnesses. The study did not collect information on breastfeeding and early childhood development though these are also important elements of HBYC.

## Conclusion

This study assessed the statewide implementation of HBYC through Mitanin CHWs in rural Chhattisgarh. The CHWs showed high coverage rates in making home visit for HBYC and delivering the necessary messages. They were able to cover a large proportion of the sick children and provide necessary management of illnesses. The CHW intervention was associated with improvement in timely initiation, frequency and diversity of complementary feeding.

Along with improving food security of households, covering a large share of young children population with quality home visits under scaled-up CHW programmes can be the key to achieving improvements in complementary feeding and child care practices in developing countries.

## Supplementary Information

**Additional file 1. **S1.

**Additional file 2. **S2.

## Data Availability

The datasets used and/or analyzed during the current study are available from the corresponding author and State Health Resource Centre, Chhattisgarh on reasonable request.

## Abbreviations

ASHA: Accredited social health activist
CF: Complementary feeding
CHWs: Community health workers
CI: Confidence interval
HBNC: Home based newborn care
HBYC: Home based care of young children
IFA: Iron and folic acid
ICDS: Integrated child development services
IYCF: Infant and young child feeding
LMICs: Low- and middle-income countries
OBC: Other backward classes
ORS: Oral rehydration solution
SC: Scheduled castes
ST: Scheduled tribes
