# Supplementary material for: Role of Mitanin community health workers in improving complementary feeding practices under scaled-up home-based care of young children in a rural region of India
Source: BMC Pediatr. 2023 Apr 13;23:171. doi: 10.1186/s12887-023-03993-4 (PMC10099942; doi:10.1186/s12887-023-03993-4)
Supplement: Supplementary file 1 — Additional file 1. S1. [file 12887_2023_3993_MOESM1_ESM.docx]

**Additional File S1**

**Table: Proportion of children who consumed various kinds of foods the previous day (in % with 95% CI)**

| **Type of food given** | **7 to 12 months (N=703) (%)** | **13 to 24 months (N=1136) (%)** | **25 to 36 months (N=807) (%)** | **Total (N=2646) (%)** |
| --- | --- | --- | --- | --- |
| Cereals | 94.3 (92.6-96.0) | 95.2 (93.9-96.4) | 95.2 (93.7-96.7) | 94.9 (94.1-95.8) |
| Pulses | 84.6 (82.0-87.3) | 81.8 (79.5-84.0) | 86.4 (84.0-88.7) | 83.9 (82.5-85.3) |
| Eggs | 17.4 (14.5-20.2) | 20.2 (17.8-22.5) | 20.2 (17.4-23.0) | 19.4 (17.9-20.9) |
| Fish/ meat | 11.7 (9.3-14.0) | 17.4 (15.2-19.6) | 17.6 (15.0-20.2) | 16.0 (14.6-17.3) |
| Vegetables | 68.6 (65.1-72.0) | 85.0 (82.9-87.0) | 86.1 (83.7-88.5) | 81.0 (79.5-82.4) |
| Fruits | 27.5 (24.1-30.8) | 34.1 (31.3-36.8) | 37.3 (34.0-40.6) | 33.3 (31.5-35.1) |
