# Supplementary material for: Role of Mitanin community health workers in improving complementary feeding practices under scaled-up home-based care of young children in a rural region of India
Source: BMC Pediatr. 2023 Apr 13;23:171. doi: 10.1186/s12887-023-03993-4 (PMC10099942; doi:10.1186/s12887-023-03993-4)
Supplement: Supplementary file 2 — Additional file 2. S2. [file 12887_2023_3993_MOESM2_ESM.docx]

**Additional File S2**

**Table: Results of adjusted models for determinants of desired complementary feeding practices**

|  | **Complementary feeding started at six months** | | **Frequency of complementary feeding** | | **Number of types of foods included in feeding on previous day** | | **Consumption of supplementary nutrition** | | **Addition of oil in child's food** | | **Weighing of child in last 2 months** | |
| --- | --- | --- | --- | --- | --- | --- | --- | --- | --- | --- | --- | --- |
| **Multivariate model** | Logistic regression | | Linear regression | | Linear regression | | Logistic regression | | Logistic regression | | Logistic regression | |
| **Number of observations (n)** | 687 | | 2606 | | 2606 | | 2606 | | 2606 | | 2606 | |
|  | **Odds Ratio** | **p** | **Coef.** | **p** | **Coef.** | **p** | **Odds Ratio** | **p** | **Odds Ratio** | **P** | **Odds Ratio** | **p** |
| **Advice received from Mitanin (Yes)** | 1.881 | 0.042 | 0.562 | <0.001 | 0.547 | <0.001 | 2.805 | <0.001 | 4.876 | <0.001 | 3.165 | <0.001 |
| **Age of child** (in months) | 1.005 | 0.935 | 0.008 | 0.117 | 0.018 | <0.001 | 0.996 | 0.448 | 0.989 | 0.027 | 0.961 | <0.001 |
| **Gender** **of child** (Reference-Male) |  |  |  |  |  |  |  |  |  |  |  |  |
| Female | 0.927 | 0.694 | 0.086 | 0.328 | -0.025 | 0.590 | 0.845 | 0.041 | 1.015 | 0.863 | 0.997 | 0.986 |
| **Mother’s Education** (Reference-8^th^ standard or higher) |  |  |  |  |  |  |  |  |  |  |  |  |
| 5-7^th^ standard | 1.556 | 0.172 | 0.119 | 0.366 | 0.065 | 0.353 | 0.980 | 0.868 | 0.914 | 0.481 | 0.799 | 0.411 |
| 1-4^th^ standard | 0.392 | 0.077 | 0.152 | 0.514 | -0.097 | 0.434 | 1.342 | 0.189 | 0.800 | 0.328 | 0.823 | 0.669 |
| No formal education | 0.507 | 0.030 | 0.132 | 0.361 | -0.152 | 0.049 | 1.156 | 0.293 | 0.973 | 0.841 | 0.682 | 0.225 |
| **Mother’s age** (in years) | 1.011 | 0.684 | -0.023 | 0.054 | -0.002 | 0.801 | 1.000 | 0.970 | 1.011 | 0.342 | 0.985 | 0.560 |
| **Family size** | 0.925 | 0.030 | -0.018 | 0.216 | -0.011 | 0.157 | 0.993 | 0.632 | 0.989 | 0.436 | 0.957 | 0.207 |
| **Social group** (Reference-Scheduled Tribes) |  |  |  |  |  |  |  |  |  |  |  |  |
| Scheduled Castes | 0.725 | 0.367 | -0.056 | 0.709 | -0.046 | 0.571 | 0.582 | <0.001 | 0.677 | 0.009 | 0.205 | <0.001 |
| Other Backward Classes | 0.604 | 0.033 | -0.323 | 0.002 | 0.028 | 0.624 | 0.897 | 0.280 | 0.954 | 0.642 | 0.354 | 0.000 |
| Others | 0.314 | 0.018 | -0.748 | <0.001 | 0.052 | 0.612 | 0.719 | 0.071 | 0.951 | 0.786 | 0.765 | 0.577 |
| **Geographical Division** (Reference-Raipur) |  |  |  |  |  |  |  |  |  |  |  |  |
| Durg | 0.433 | 0.004 | -0.078 | 0.572 | -0.304 | <0.001 | 0.868 | 0.270 | 0.957 | 0.739 | 0.393 | 0.020 |
| Bilaspur | 0.945 | 0.849 | 0.502 | <0.001 | 0.047 | 0.499 | 0.582 | 0.000 | 0.903 | 0.422 | 0.643 | 0.288 |
| Sarguja | 0.657 | 0.205 | 0.194 | 0.196 | 0.026 | 0.747 | 1.096 | 0.518 | 0.865 | 0.317 | 0.081 | <0.001 |
| Bastar | 0.475 | 0.029 | 0.411 | 0.010 | -0.171 | 0.044 | 1.342 | 0.053 | 1.148 | 0.364 | 0.309 | 0.011 |
